# Supplementary material for: Iterative improvement in the automatic modular design of robot swarms
Source: PeerJ Comput Sci. 2020 Dec 7;6:e322. doi: 10.7717/peerj-cs.322 (PMC7924708; doi:10.7717/peerj-cs.322)
Supplement: Supplemental Information 3 [file peerj-cs-06-322-s003.zip › argos3/doc/api/standalone/a00594_source.html]

ARGoS: plugins/simulator/entities/wifi\_equipped\_entity.cpp Source File


- Main Page
- Related Pages
- Namespaces
- Classes
- Files

- File List
- File Members

# plugins/simulator/entities/wifi\_equipped\_entity.cpp

Go to the documentation of this file.

```
00001 
00007 #include "wifi_equipped_entity.h"
00008 #include <argos3/core/simulator/space/space.h>
00009 
00010 namespace argos {
00011 
00012    /****************************************/
00013    /****************************************/
00014 
00015    CWiFiEquippedEntity::CWiFiEquippedEntity(CComposableEntity* pc_parent) :
00016       CEntity(pc_parent) {
00017       Disable();
00018    }
00019 
00020    /****************************************/
00021    /****************************************/
00022 
00023    CWiFiEquippedEntity::CWiFiEquippedEntity(CComposableEntity* pc_parent,
00024                                             const std::string& str_id) :
00025       CEntity(pc_parent, str_id) {
00026       Disable();
00027    }
00028 
00029    /****************************************/
00030    /****************************************/
00031 
00032    REGISTER_STANDARD_SPACE_OPERATIONS_ON_ENTITY(CWiFiEquippedEntity);
00033 
00034    /****************************************/
00035    /****************************************/
00036 
00037 }
```

---

Generated on 10 Jul 2018 for ARGoS by 
 1.6.1
